# British Surgery in Paris

**Published:** 1864-03

**Authors:** 


British Surgery in Paris.—At a late meeting of the Surgical Society of Paris, three well-known surgeons belonging to this country were unanimously elected foreign associates, viz: Mr. Robert Adams, of Dublin, Mr. Joseph Hodgson, and Mr. James Paget, both of London.—*London Lancet.*

